# A Case Report of Diffuse Alveolar Hemorrhage Coexisting With Immunoglobulin A (IgA) Nephropathy

**DOI:** 10.7759/cureus.31941

**Published:** 2022-11-27

**Authors:** Michael Kolman, Shen Song, Tina Han, Nahren Asado, Eli D Ehrenpreis

**Affiliations:** 1 Internal Medicine, Advocate Lutheran General Hospital, Park Ridge, USA; 2 Nephrology, Advocate Lutheran General Hospital, Park Ridge, USA; 3 Pathology, Advocate Lutheran General Hospital, Park Ridge, USA; 4 Internal Medicine, Advocate Lutheran General Hospital, Skokie, USA

**Keywords:** chronic hepatitis b infection, pulmonary-renal syndrome, henoch-schönlein purpura (iga vasculitis), diffuse alveolar hemorrhage, iga nephropathy

## Abstract

Immunoglobulin A (IgA) nephropathy is the most common cause of primary glomerulonephritis worldwide. IgA vasculitis (formerly known as Henoch-Schonlein purpura) typically presents with IgA nephropathy on renal biopsy in addition to extrarenal symptoms like purpura, abdominal pain, and arthritis. Diffuse alveolar hemorrhage (DAH) is the most common pulmonary complication, but this is rarely seen. In this case report, we describe a 35-year-old male with chronic untreated hepatitis B infection who presented with pulmonary-renal syndrome. He was found to have clinical findings of DAH and concomitant IgA nephropathy on renal biopsy, without having any other typical manifestations of IgA vasculitis. This shows that IgA nephropathy should be considered in the differential diagnosis of DAH and emphasizes the importance of a renal biopsy in patients presenting with pulmonary-renal syndrome.

## Introduction

Immunoglobulin A (IgA) nephropathy is a condition in which immune complexes deposit in the small blood vessels of the kidney [1]. IgA vasculitis (formerly known as Henoch-Schonlein purpura) typically causes IgA nephropathy and commonly affects the skin, gastrointestinal tract, and joints [2]. It is rare to have pulmonary symptoms, although diffuse alveolar hemorrhage (DAH) has been associated with the disease [3]. The most common trigger is a recent upper respiratory infection [4]. This case report is about a young man with a history of untreated hepatitis B who was found to have clinical manifestations of DAH and renal biopsy consistent with IgA nephropathy without other extrarenal symptoms, which may be seen as an unusual presentation of IgA vasculitis. This demonstrates that a renal biopsy should be considered in patients with pulmonary-renal syndrome.

## Case presentation

A 35-year-old male with a past medical history of untreated chronic hepatitis B presented to the emergency department (ED) with hemoptysis. The patient reported that he had a chronic dry cough for approximately four to five weeks. The night prior to coming into the hospital, he noticed frank blood in his coughs. He also endorsed right foot swelling that he took Aleve for, in addition to pleuritic chest pain when the hemoptysis began. He denied fever, chills, night sweats, weight loss, nausea, vomiting, diarrhea, body aches, arthralgia, and rashes.

**Figure 1:**
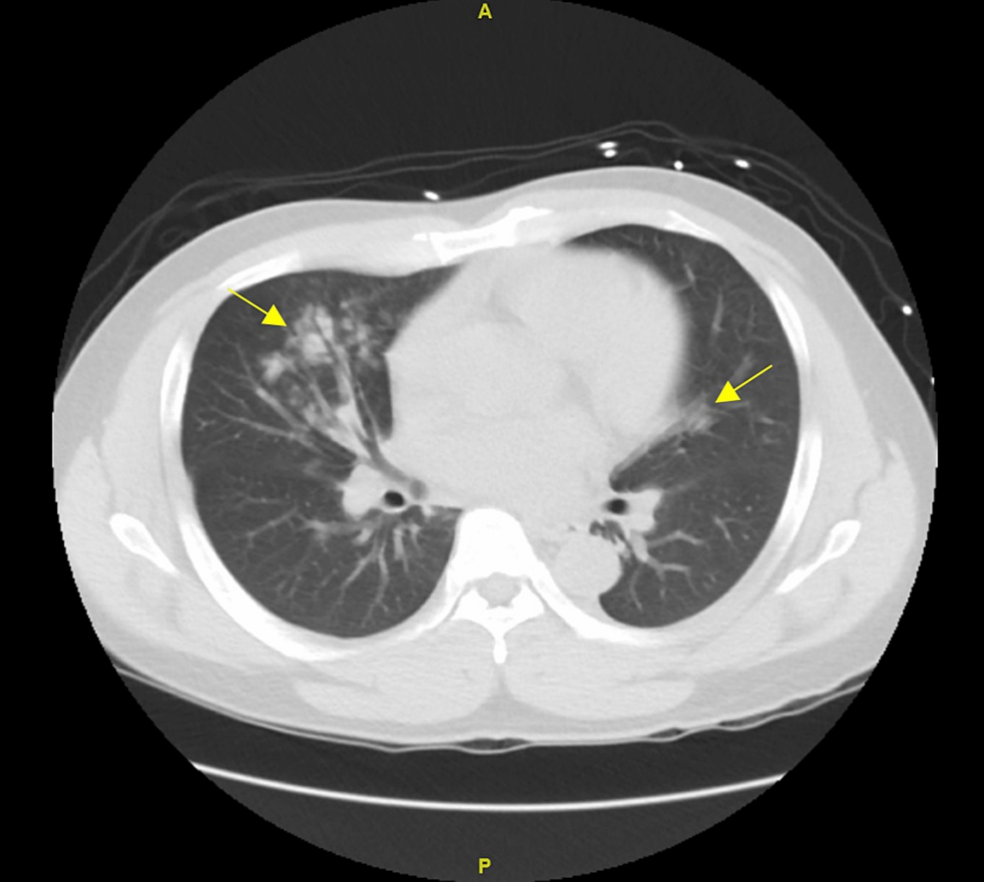
Scattered clusters of centrilobular ground-glass nodules bilaterally (yellow arrows), worse on the right

The patient was born in the Philippines, moved to the United States seven years ago, and has not traveled recently. He worked as a caregiver, and to his knowledge, had not taken care of any patients with a known infectious disease. He had no history of autoimmune disease. He did not use tobacco, was a social alcohol drinker, and did not do any recreational drugs. In the ED, the patient was afebrile, with a blood pressure of 167/114 mmHg, heart rate of 79 beats per minute, respiratory rate of 18 breaths per minute, and O_2_ saturation of 94% on room air. Physical examination was only significant for mildly decreased breath sounds in the lower lung bases. The basic metabolic panel was significant for sodium 136 mEg/L, potassium 5.8 mEq/L, blood urea nitrogen (BUN) 77 mg/dL, and creatinine 4.88 mg/dL with an unknown baseline (Table 1). Complete blood count was significant for white blood cell count (WBC) 12.1 K/mcL, red blood cell count (RBC) 3.82 mil/mcL, hemoglobin 10.8 g/dL, mean corpuscular volume (MCV) 87.2 fl, and platelets 231 K/mcL (Table 2). Urinalysis showed 30 mg/dL protein and few bacteria, but no blood was noted (Table 3). As the patient had abnormal creatinine and there was a concern for glomerulonephropathy, his urine protein/creatinine ratio was checked and was reported as 3.068 gPR/gCR (Table 4). Computerized tomography (CT) of the chest without contrast showed scattered clusters of centrilobular ground-glass nodules bilaterally, which were worse on the right (Figure 1).

**Table 1:** Complete metabolic panel mEq/L = milliequivalents per liter; mmol/L = millimoles per liter; mg/dL = milligrams per deciliter; g/dL = grams per deciliter; Units/L = units per liter

COMPLETE METABOLIC PANEL	RESULTING LABORATORY VALUE	REFERENCE RANGE
SODIUM	136 mEq/L	135 – 145 mmol/L
POTASSIUM	5.8 mEq/L	3.4 – 5.1 mmol/L
CHLORIDE	109 mmol/L	98 – 107 mmol/L
BICARBONATE	22 mmol/L	21 – 32 mmol/L
BLOOD UREA NITROGEN	77 mg/dL	6 – 20 mg/dL
CREATININE	4.88 mg/dL	0.67 – 1.17 mg/dL
GLUCOSE	103 mg/dL	70 – 99 mg/dL
CALCIUM	9.0 mg/dL	8.4 – 10.2 mg/dL
BILIRUBIN, TOTAL	0.5 mg/dL	0.2 – 1.0 mg/dL
ASPARTATE AMINOTRANSFERASE (AST)	27 Units/L	<=37 Units/L
ALANINE TRANSAMINASE (ALT)	48 Units/L	<64 Units/L
ALKALINE PHOSPHATASE	98 Units/L	45 – 117 Units/L
ALBUMIN	3.5 g/dL	3.6 – 5.1 g/dL
PROTEIN, TOTAL	7.3 g/dL	6.4 – 8.2 g/dL
GLOBULIN	4.7 g/dL	2.0 – 4.0 g/dL

**Table 2:** Complete blood count K/mcL = thousands of cells per microliter of blood; mil/mcL = million cells per microliter; g/dL = grams per deciliter; fl = femtoliters; pg = picograms

COMPLETE BLOOD COUNT	RESULTING LABORATORY VALUE	REFERENCE RANGE
WHITE BLOOD CELLS (WBC)	12.1 K/mcL	4.2 – 11.0 K/mcL
RED BLOOD CELLS (RBC)	3.82 mil/mcL	4.50 – 5.90 mil/mcL
HEMOGLOBIN (HGB)	10.8 g/dL	13.0 – 17.0 g/dL
MEAN CORPUSCULAR VOLUME (MCV)	87.2 fl	78.0 – 100.0 fl
MEAN CORPUSCULAR HEMOGLOBIN (MCH)	28.3 pg	26.0 – 34.0 pg
PLATELETS	231 K/mcL	140 – 450 K/mcL

**Table 3:** Urinalysis hpf = high power field

URINALYSIS	RESULTING LABORATORY VALUE	REFERENCE RANGE
COLOR, URINALYSIS	STRAW	REFERENCE RANGE NOT AVAILABLE
APPEARANCE, URINALYSIS	CLEAR	REFERENCE RANGE NOT AVAILABLE
OCCULT BLOOD, URINALYSIS	NEGATIVE	NEGATIVE
PROTEIN, URINALYSIS	30	NEGATIVE
NITRITE, URINALYSIS	NEGATIVE	NEGATIVE
LEUKOCYTE ESTERASE, URINALYSIS	NEGATIVE	NEGATIVE
LEUKOCYTES, URINALYSIS	1 TO 5 / hpf	NONE SEEN, 1 TO 5 / hpf
ERYTHROCYTES, URINALYSIS	1 TO 2 / hpf	NON SEEN, 1 TO 5 / hpf
BACTERIA, URINALYSIS	NONE SEEN	NON SEEN / hpf

**Table 4:** Protein/creatinine ratio, urine mg/dL = milligrams per deciliter; mgPR/gCR = milligrams protein per grams creatinine

PROTEIN/CREATININE RATIO, URINE	RESULTING LABORATORY VALUE	REFERENCE RANGE
PROTEIN, URINE	139 mg/dL	< 12 mg/dL
CREATININE, URINE	45.30 mg/dL	REFERENCE RANGE NOT AVAILABLE
PROTEIN/CREATININE RATIO	3,068 mgPR/gCR	<=199 mgPR/gCR

The nephrology and infectious disease services were consulted. The patient was started on IV Solu-Medrol 500 mg for three days, as there was a concern for diffuse alveolar hemorrhage secondary to vasculitis. The patient's hemoptysis resolved quickly, but renal function was slow to downtrend. His elevated creatinine was thought to be due to glomerulonephritis in the setting of vasculitis, but other differentials included membranous nephropathy with the patient's untreated hepatitis B or acute kidney injury due to Aleve use. The patient was found to be positive for hepatitis B surface antigen and hepatitis B core antibody. His hepatitis B quantitative DNA was 9850 IU/mL (Table 5). The patient was started on Entecavir for hepatitis B. Further laboratory workup demonstrated an erythrocyte sedimentation rate (ESR) of 72 mm/hr and C-reactive protein (CRP) of 7.8 mg/dL. Antinuclear antibodies (ANA) were positive, but the titer was <1:80 (Table 6). Pertinent negative labs throughout the admission included troponin, SARS-CoV-2 RNA, Quant TB, myeloperoxidase antibody, proteinase-3 antibody, glomerular basement membrane IgG antibody, HIV, Histoplasma urine antigen, Aspergillus galactomannan antigen. The patient's C3 and C4 complement levels were within normal limits (Table 6).

**Table 5:** Hepatitis panel IU/mL = International unit per milliliter

HEPATITIS PANEL	RESULTING LABORATORY VALUE	REFERENCE RANGE
HEPATITIS A ANTIBODY, IGM	NEGATIVE	NEGATIVE
HEPATITIS B CORE ANTIBODY, IGG	POSITIVE	NEGATIVE
HEPATITIS B CORE ANTIBODY, IGM	NEGATIVE	NEGATIVE
HEPATITIS B SURFACE ANTIGEN	POSITIVE	NEGATIVE
HEPATITIS BE ANTIBODY	POSITIVE	NEGATIVE
HEPATITIS BE ANTIGEN	NEGATIVE	NEGATIVE
HEPATITIS B VIRUS, QUANTITATIVE	9,850 IU/mL	< = 0.0 IU/mL
HEPATITIS C ANTIBODY	NEGATIVE	NEGATIVE

**Table 6:** Inflammatory markers and infectious organisms mg/dL = milligrams per deciliter; mm/hr = millimeters per hour; ng/L = nanograms per liter

INFLAMMATORY MARKER OR INFECTIOUS ORGANISM	RESULTING LABORATORY VALUE	REFERENCE RANGE
ANTINUCLEAR ANTIBODIES	POSITIVE, <1:80	<1:80
COMPLEMENT C3	100 mg/dL	79 – 152 mg/dL
COMPLEMENT C4	19.9 mg/dL	16.0 – 38.0 mg/dL
MYELOPEROXIDASE ANTIBODY	<0.2	<1.0
PROTEINASE-3 ANTIBODY	<0.2	<1.0
GLOMERULAR BASEMENT MEMBRANE, IGG ANTIBODY (ANTI-GBM)	<0.2	<1.0
C-REACTIVE PROTEIN (CRP)	7.8 mg/dL	<1.0 mg/dL
ERYTHROCYTE SEDIMENTATION RATE (ESR)	72 mm/hr	0 – 20 mm/hr
TROPONIN, HIGH SENSITIVITY	64 ng/L	<77 ng/L
ASPERGILLUS GALACTOMANNAN ANTIGEN	NEGATIVE	NEGATIVE
HISTOPLASMA ANTIGEN, URINE	NOT DETECTED	NOT DETECTED
SARS COV 2 RNA	NOT DETECTED	NOT DETECTED
HIV ANTIGEN/ANTIBODY SCREEN	NONREACTIVE	NONREACTIVE
QUANTIFERON PLUS INTERPRETATION	NEGATIVE	NEGATIVE

**Figure 2:**
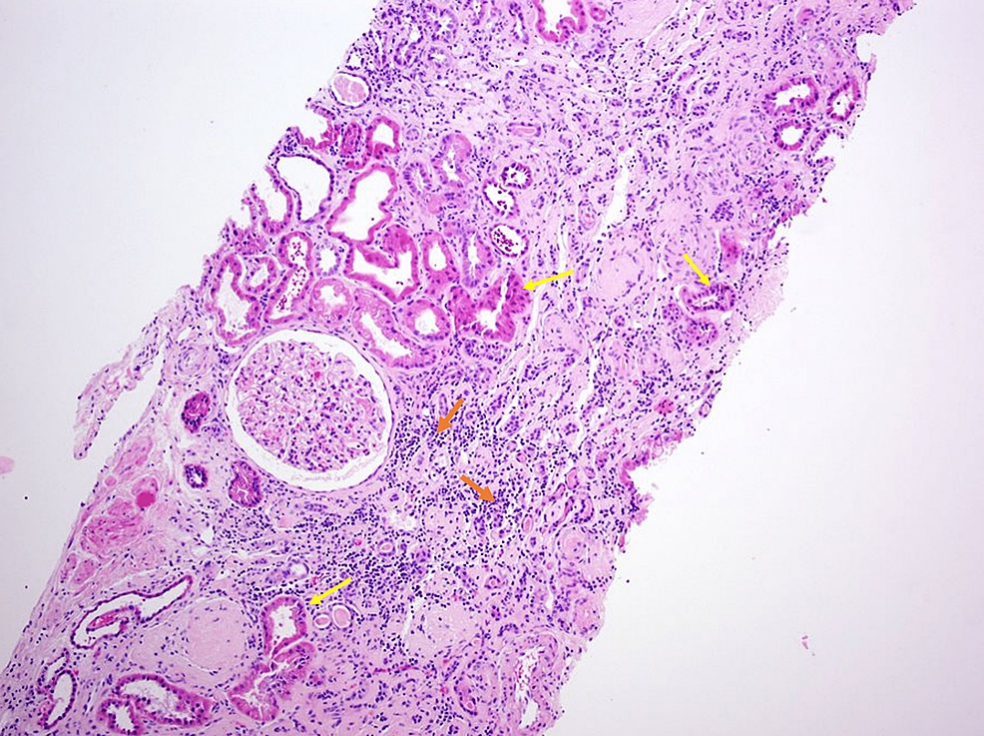
Renal cortical tissue showing marked chronic tubulointerstitial changes. Lymphocytes are seen in the tubular area (yellow arrows) and in the interstitium (orange arrows). H&E stain. 10x.

**Figure 3:**
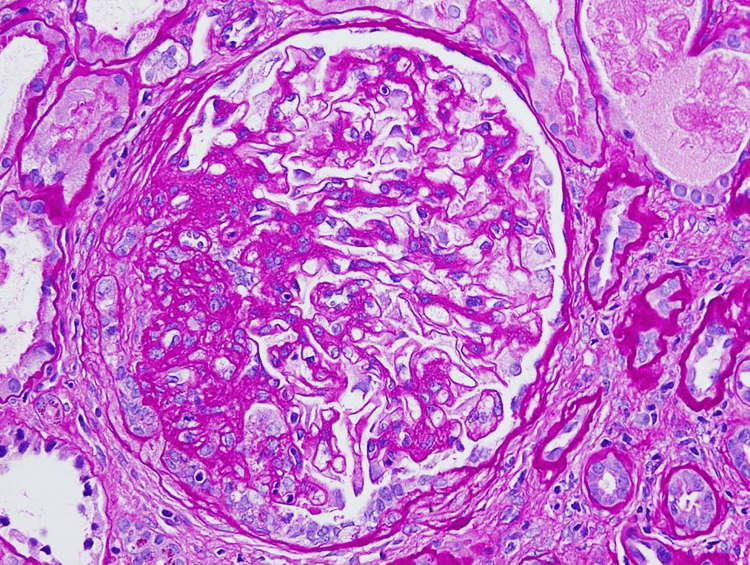
Enlarged glomerulus showing mild mesangial expansion and a segmental sclerotic lesion with overlying epithelial cell hyperplasia periodic-acid-Schiff (PAS) stain. 40x.

**Figure 4:**
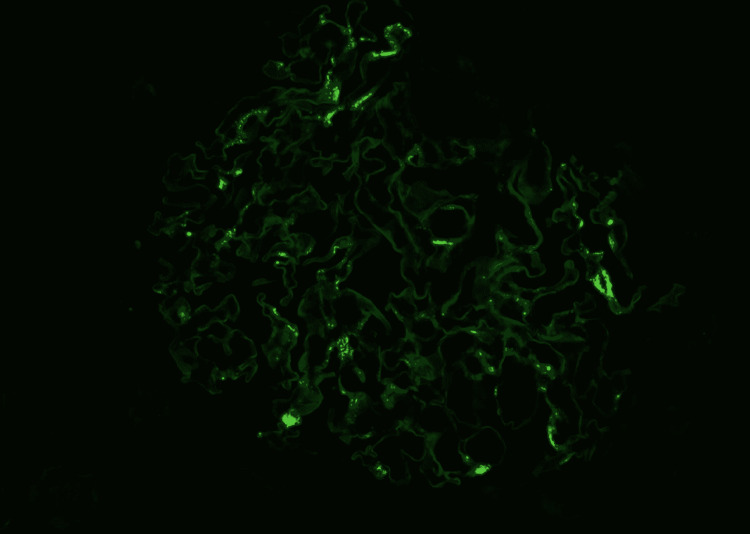
Immunofluorescence examination revealing granular mesangial deposits of IgA IgA: immunoglobulin A

**Figure 5:**
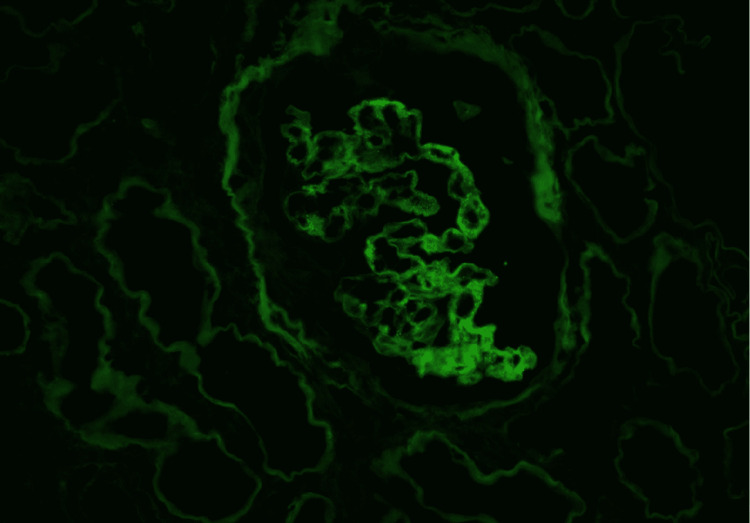
Immunofluorescence examination revealing mesangial deposits of light chain lambda

**Figure 6:**
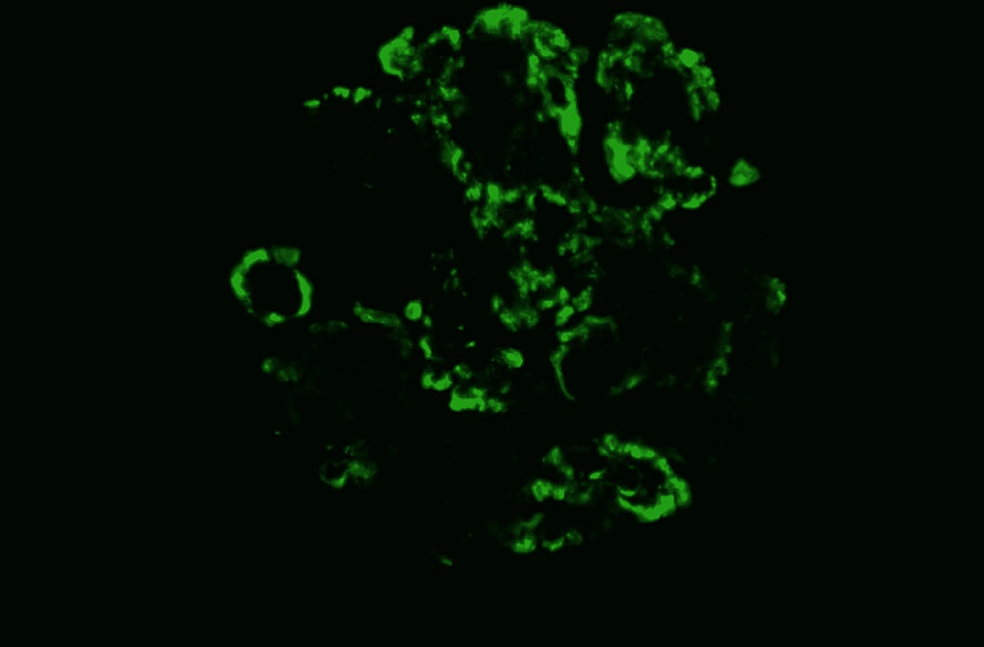
Immunofluorescence examination revealing segmental glomerular staining of C3, faint mesangial staining

**Figure 7:**
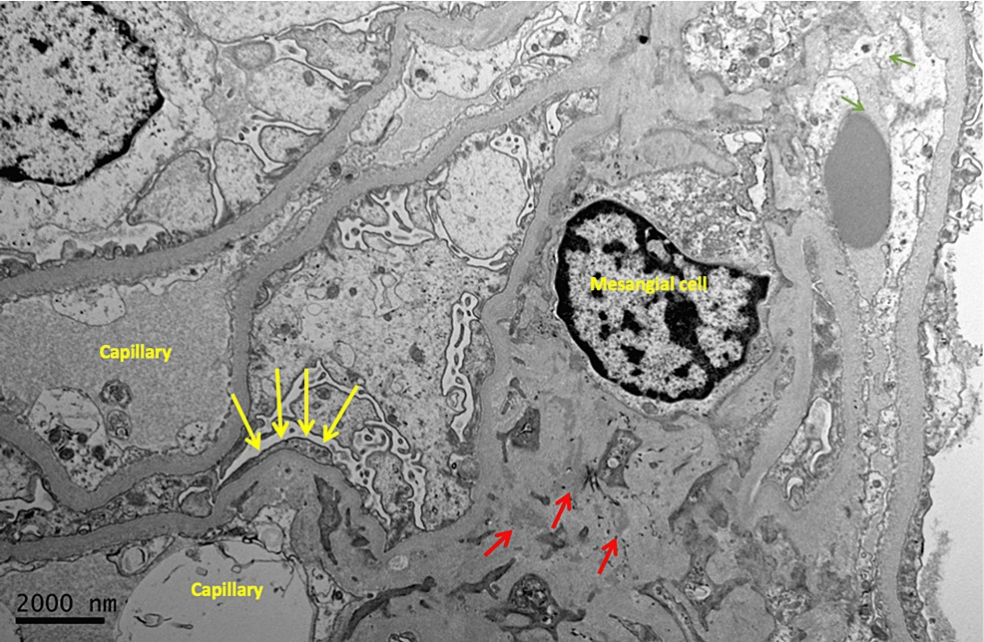
Electron microscopic image revealing mesangial expansion with abundant mesangial electron-dense deposits (red arrows) and segmental attenuated podocyte foot processes (yellow arrows) 2000 nm

The patient had a renal biopsy, which demonstrated IgA nephropathy, diffuse global glomerulosclerosis, and chronic tubulointerstitial nephritis. Seventy-two percent (72%) of sampled glomeruli were globally sclerosed and 17% with segmental sclerosis, also with significant chronic hypertension (HTN) changes (Figures 2-7).

On discharge, the patient did not have hemoptysis for over 48 hours and his creatinine down-trended to 4.39. As no crescents were seen on renal biopsy and the patient was clinically improving, the nephrology team on the case reported there was no further indication for steroids. He was instructed to follow up closely with the nephrology team outpatient to monitor his creatinine and see what his baseline is prior to starting an angiotensin-converting enzyme (ACE) inhibitor or angiotensin receptor blocker (ARB) to slow the progression of chronic kidney disease and tighten blood pressure control.

## Discussion

This case describes an adult male with presumed DAH in the setting of IgA nephropathy without having any other symptoms consistent with IgA vasculitis.

The diagnosis of DAH is suspected in patients with hemoptysis, anemia, progressive dyspnea, and a pattern of focal or diffuse areas of ground glass opacities or consolidations on chest imaging [5-7]. The most common causes are systemic vasculitides, connective tissue disorders, immune-complex-mediated disorders, and post-transplant hemorrhages [5]. Early bronchoscopy with bronchoalveolar lavage is the diagnostic gold standard for DAH, as the accumulation of blood can be seen in the alveolar spaces and infection can be more definitively ruled out [7]. Treatment consists of starting high-dose corticosteroids promptly to reduce inflammation and lung alveolar epithelial swelling [5].

Per the infectious disease team taking care of this patient, DAH was the most likely etiology of the patient’s pulmonary symptoms, as infectious workup was negative and he had symptoms and imaging findings consistent with the disease, as noted above. The patient was immediately started on IV Solu-Medrol 500 mg for three days for concern of pulmonary-renal syndrome. Since he did not have any further episodes of hemoptysis and was breathing well on room air, a bronchoscopy was not done.

Over the course of the hospitalization, the patient had a renal biopsy done, which showed IgA nephropathy. The differential diagnosis for pulmonary-renal syndrome in the setting of IgA nephropathy includes IgA nephropathy-related DAH, IgA vasculitis (formerly known as Henoch-Schoenlein purpura), IgA nephropathy-antineutrophil cytoplasmic antibodies (ANCA) vasculitis overlap syndrome, IgA-dominant post-infectious glomerulonephritis, and IgA-variant Goodpasture's syndrome [8]. As the patient's ANCA was negative, this made IgA nephropathy-ANCA vasculitis overlap syndrome less likely. While the IgA antibody against the glomerular basement membrane was not tested in this patient, immunofluorescence microscopy did not show linear deposition along the glomerular basement membrane, making IgA-variant Goodpasture's syndrome unlikely to be the diagnosis. It is also unlikely that the patient had post-infectious glomerulonephritis, as this often manifests after resolution of bacterial infection (Group A beta-hemolytic Streptococcus infection being the most common), infective endocarditis, or VP shunt nephritis [9], none of which the patient had. This leaves IgA nephropathy-related DAH and IgA vasculitis at the top of the differential diagnoses for this patient's presentation.

The renal symptoms in IgA nephropathy and IgA vasculitis are virtually indistinguishable [8]. The main differences are that IgA vasculitis typically also presents with skin manifestations (purpura), GI manifestations (abdominal pain), and arthralgias [2], none of which were present in this patient. In IgA vasculitis, it is rare to have pulmonary involvement, although DAH is the most common form [3]. In many cases, IgA vasculitis occurs after an upper respiratory tract infection but certain medications, insect bites, immunizations, and foods have been associated with the disease as well [4]. Lastly, over 90% of patients with IgA vasculitis are children, with nephritis being the most common complication seen in adults [10].

IgA nephropathy is thought to develop when there is abnormal glycosylation of the serine and threonine residues of the IgA-1 hinge region. These galactose-deficient IgA-1 antibodies are initially not identified by the body, and therefore not degraded, allowing them to accumulate. Over time, the body generates anti-glycan IgG antibodies to target these galactose-deficient IgA-1 antibodies. When these anti-glycan IgG antibodies bind to the galactose-deficient IgA-1 antibodies, immune complexes form, which deposit in various parts of the body, including small blood vessel walls and the mesangium of the kidney [1].

Although it is unknown what the trigger was for this patient’s pulmonary-renal syndrome, it is thought that the patient’s chronic hepatitis B infection could have contributed. There are case reports that have suggested chronic hepatitis B plays a role in the development of IgA nephropathy in an indolent, progressive manner [11], but there has been minimal documentation on it contributing to IgA vasculitis or a pulmonary-renal syndrome involving IgA nephropathy.

There have been a few case reports and systemic reviews on DAH in the setting of IgA nephropathy, but there is no clear consensus on its management. One study reported that the treatment with oral corticosteroids alone was equally as effective as IV pulse corticosteroids alone, but combination therapy with a corticosteroid and immunosuppressive was most effective [8]. Whereas a different report mentioned that there was no difference in renal outcomes and mortality when comparing corticosteroids alone versus combination therapy of corticosteroids with an immunosuppressive such as cyclophosphamide [2]. The nephrology team on the case for this patient made the decision to avoid further use of corticosteroids or immunosuppressives after the pulse dose IV Solu-Medrol, as there is no clear consensus on the management of DAH in the setting of IgA nephropathy, the patient’s hemoptysis resolved, creatinine down-trended, and renal biopsy showed signs of chronic tubulointerstitial nephritis without crescents.

## Conclusions

In conclusion, this case highlights two unique findings. While many extrarenal manifestations have been described in IgA nephropathy, pulmonary involvement is very rare. Second, this case demonstrates that IgA nephropathy should be considered in the differential diagnosis of DAH. These combined findings emphasize the importance of obtaining a renal biopsy in the setting of pulmonary-renal syndrome to prevent delay of proper diagnosis and treatment.
